# Cost-effectiveness of neck-specific exercise with or without a behavioral approach versus physical activity prescription in the treatment of chronic whiplash-associated disorders: Analyses of a randomized clinical trial

**DOI:** 10.1097/MD.0000000000007274

**Published:** 2017-06-23

**Authors:** Maria Landén Ludvigsson, Anneli Peolsson, Gunnel Peterson, Åsa Dedering, Gun Johansson, Lars Bernfort

**Affiliations:** a Division of Physiotherapy, Department of Medical and Health Sciences; b Rehab Väst, County Council of Östergötland, Departments of Rehabilitation and Medical and Health Sciences; c Centre for Clinical Research Sörmland, Uppsala University; d Allied Health Professionals Function, Karolinska University Hospital; e Division of Physiotherapy, Department of Neurobiology, Care Sciences and Society, Karolinska Institutet, Huddinge; f Karolinska Institutet, Institute of Environmental Medicine, Unit of Occupational Medicine, Stockholm; g Division of Health Care Analysis, Department of Medical and Health Sciences, Linköping University, Sweden.

**Keywords:** chronic, cost, cost-effectiveness, exercise, physiotherapy, rehabilitation, whiplash

## Abstract

**Background:**

Fifty percent of people injured by whiplash still report neck pain after 1 year and costs associated with whiplash associated disorders (WAD) are mostly attributed to health service and sick-leave costs in chronic conditions. With increasing health care expenditures the economic impact of interventions needs to be considered.

**Objective:**

To analyze the cost-effectiveness of physiotherapist-led neck-specific exercise without (NSE) or with a behavioral approach (NSEB), or prescription of physical activity (PPA) in chronic WAD, grade 2 to 3.

**Methods:**

This is a secondary cost-effectiveness analysis of a multicenter randomized clinical trial of 216 participants with chronic WAD grade 2 to 3. The interventions were physiotherapist-led neck-specific exercise without or with a behavioral approach, or prescription of physical activity for 12 weeks. Incremental cost-effectiveness ratios (ICERs) were determined after 1 year and bootstrapped cost-effectiveness planes and sensitivity analyses of physiotherapy visits were performed. Health care and production loss costs were included and quality-adjusted life years (QALYs) were estimated, using the Euroqol-5D questionnaire. Comparisons with the Short Form-6D, and neck disability index (NDI) were also made.

**Results:**

The 1-year follow-up was completed by 170 participants (79%). Both physiotherapist-led groups improved in health related quality of life. The intervention cost alone, per quality-adjusted life year (QALY) gain in the NSE group was US$ 12,067. A trend for higher QALY gains were observed in the NSEB group but the costs were also higher. The ICERs varied depending on questionnaire used, but the addition of a behavioral approach to neck-specific exercise alone was not cost-effective from a societal perspective (ICER primary outcome $127,800 [95% confidence interval [CI], 37,816–711,302]). The sensitivity analyses confirmed the results. The prescription of physical activity did not result in any QALY gain and the societal costs were not lower.

**Conclusion:**

Neck-specific exercise was cost-effective from a societal perspective in the treatment of chronic WAD compared with the other exercise interventions. ICERS varied depending on health-related quality of life questionnaires used, but the addition of a behavioral approach was not cost-effective from a societal perspective. The prescription of physical activity did not result in any QALY gain and was thus not considered a relevant option.

## 1 Introduction

Whiplash injuries have a broad effect on society in terms of health, productivity, and costs.^[1]^ With increasing health care expenditures and limited resources, the economic impact of interventions should also be considered. Hospital visits due to whiplash-associated disorders (WAD) have increased over the past few decades, and the annual incidence of reported whiplash injuries is likely to be at least 300 per 100,000.^[1]^ Impairment and disability due to WAD have also increased.^[2,3]^ Fifty percent of people injured by whiplash still report neck pain 1 year after the injury.^[4]^ In the UK, 1500 whiplash claims are made daily,^[5]^ and many require health care. The median number of physiotherapy sessions per person in Australia due to WAD is reportedly relatively high (15 sessions).^[6]^ Yet a recent review of the literature failed to find any cost-effectiveness evaluations of treatments for chronic WAD.^[7]^ WAD are estimated to cost approximately US $4 billion (£3.1 billion) per year in the UK.^[2]^ Costs associated with WAD are mostly attributed to health service costs for people with chronic (>6 months)^[8]^ symptoms and to the subsequent loss of work.^[2,3]^

Both physical and psychosocial factors have been attributed to the persistence of symptoms in people with WAD, and numerous studies report characteristic morphological changes and altered cervical muscle behavior in people with WAD.^[9–13]^ Exercise is often recommended but there is no clear evidence of effective treatment.^[14]^ In chronic WAD grade 1 to 2 (1  =  without physical signs, 2  =  local physical neck signs),^[8]^ a booklet and advice from a physiotherapist is reportedly equally effective as a more comprehensive physiotherapy exercise program.^[15]^ However, in chronic WAD grade 2 to 3 (3  =  local plus neurological signs) both significant and clinically relevant improvements have been found following physiotherapist-led neck-specific exercise, with or without a behavioral approach, compared with the prescription of physical activity outside the health care system.^[16,17]^ This result may be due to the partly different interventions and WAD groups, as WAD grade 3 has been associated with responsiveness to exercise.^[18]^ The neck-specific exercises included activation of the deep cervical muscles, and the behavioral approach was aimed at pain management.^[16]^ However, whether the higher cost of physiotherapist-led exercises compared with a prescription of physical activity is justified needs to be determined. In acute WAD, individually tailored physiotherapy is reportedly not cost-effective compared with usual care from a health care perspective,^[3]^ but to the best of our knowledge cost-effectiveness has not been reported for the treatment of chronic WAD.

Having a whiplash injury is 1 factor that predicts reduced health-related quality of life (HRQoL).^[19]^ In cost-utility analyses, generic HRQoL measurements such as the Euroqol 5-D (EQ-5D)^[20]^ and the Short Form 6D (SF-6D)^[21]^ are traditionally used. However, they do not provide interchangeable utility estimates due to contextual differences and the number of available response options.^[22]^ The SF-6D is better able to detect small changes and is more sensitive to changes in higher scores, whereas the EQ-5D is more sensitive to changes in lower scores.^[23]^ A neck-specific measurement, the neck disability index (NDI) has also been suggested as a relevant option since disease specific instruments are often more sensitive.^[24]^

The research question of this study was: Is physiotherapist-led neck-specific exercise with or without a behavioral approach or prescription of physical activity cost-effective from a societal perspective in chronic WAD, grade 2 and 3, and does the choice of health-related quality of life measure matter?

## 2 Method

### 2.1 Design

This is a secondary cost-effectiveness analysis with a 1- year follow-up of a multicenter prospective randomized clinical trial with group allocation blinding. The study was approved by The Ethics Committee of Linköping University.

### 2.2 Participants, therapists, centers

**Figure 1:**
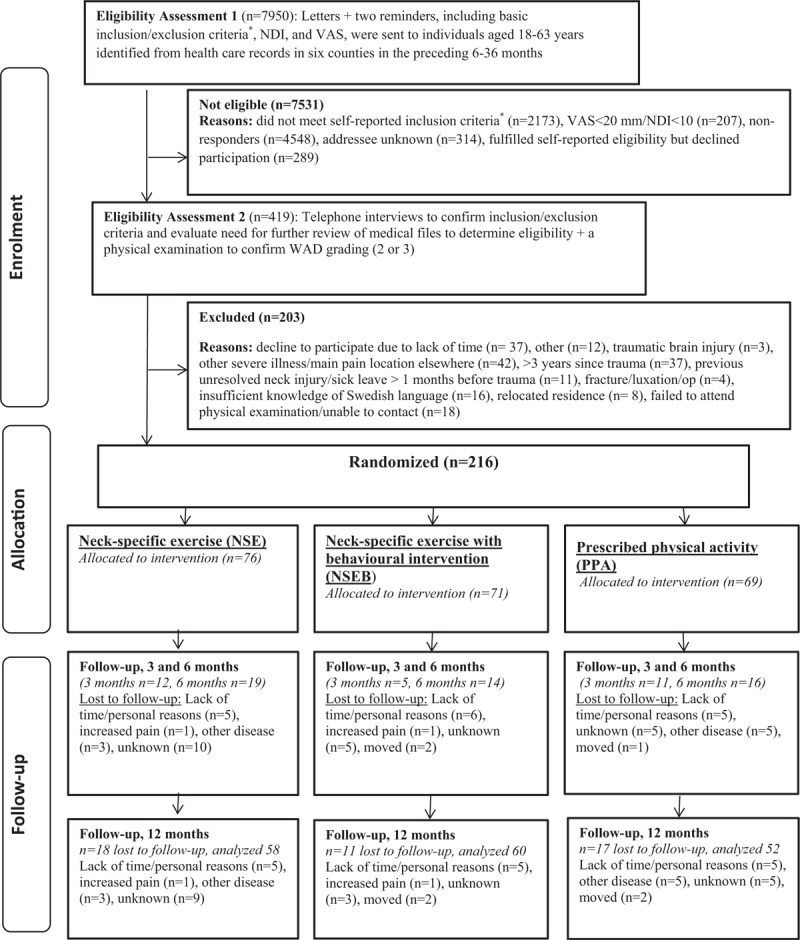
Flow-chart. ^∗^Whiplash injury in the preceding 6 to 36 months, reported to be the onset of current symptoms, excluding unconsciousness/loss of memory in connection to the whiplash injury, previous neck trauma with unresolved, symptoms, previous neck surgery, ongoing malignant disease, severe psychiatric disorders, drug abuse, difficulties understanding the Swedish language. NDI  =  neck disability index, VAS  =  visual analogue scale, WAD  =  whiplash-associated disorders.

A total of 216 individuals with chronic WAD were recruited in 2011 to 2012, including 142 (65%) women and 74 (35%) men, with a mean age of 40.5 (range 18–63, SD 11.4) years. The inclusion criteria were age 18 to 63 years, a whiplash injury, grade 2 or 3, in the preceding 6 to 36 months, nominated as the cause of current symptoms, and an NDI score of >10/50 points^[25]^ and/or an average pain >20/100 mm on a visual analogue scale (VAS) (0  =  no pain, 100  =  worst imaginable pain) for the preceding week. Exclusion criteria can be found in Figure 1 and previous publications.^[16,17]^ Potential participants were identified from health care registers and were screened for eligibility by a 4-step process where the final step was a physical examination confirming that the criteria for WAD grades 2 and 3 were met (Fig. 1 and further descriptions in Ludvigsson et al^[16]^). This test was also repeated at 3, 6, and 12 months. Informed consent and baseline outcome measurements were collected before allocation, which was made from a computer-generated randomization list by an independent researcher otherwise not involved in the study, who also sealed individual, completely opaque envelopes that were sent to the treating physiotherapists. All 3 interventions were conducted by experienced physiotherapists in primary care, chosen to match their knowledge and interest with the interventions as far as possible, in 6 Swedish counties.

### 2.3 Interventions

The interventions were: physiotherapist-led neck-specific exercise (NSE), Neck-specific exercise with the addition of a behavioral approach (NSEB) NSE with the addition of a behavioral approach (NSEB), or prescription of physical activity (PPA) which was performed over a 12-week period. In the NSE and NSEB groups, participants were scheduled for supervised neck-specific exercise twice weekly. The estimated mean time for NSE was 30 min/session and 40 minutes for the NSEB group. After individual manual guidance to ensure activation of the deeper neck muscles in the NSE/NSEB groups, exercise was performed in the gym with head resistance training, focusing on low load endurance. A detailed description of the exercises can be found at the Academic Archive On-line.^[26]^ Exercise-related pain was avoided in the NSE group, but in accordance with the concept of graded exercise, patients in the NSEB group were encouraged not to focus on temporary increases in neck pain. A behavioral approach that included education and introduction to activities aimed at pain management and problem-solving was also added in the NSEB group. In the PPA group, participants initially underwent a short motivational interview by a physiotherapist. Based on these discussions and a physical examination (total 1 hour session), the participants were prescribed individualized physical activity (e.g., Nordic walking, gym classes, etc.) to be performed independently either at home or elsewhere outside the health care system. One follow-up visit or phone call was encouraged. Participants were encouraged to continue exercising independently after the interventions in all 3 groups. Timeframes and specific components of the interventions have been described previously.^[16]^

### 2.4 Outcomes

#### 2.4.1 Cost-utility

To provide a generic measure for comparing health-related outcomes between treatments, quality adjusted life-years (QALYs) were calculated. One QALY equals 1 year in perfect health, and cost-utility is expressed as cost per QALY gained. Incremental cost-effectiveness ratios (ICERs) represent comparisons between groups of the incremental costs associated with one additional QALY and are calculated as the ratio of the cost of treatment X minus the cost of treatment Y/effect of treatment X minus the effect of treatment Y. The main outcome for this study was the ICER from a Swedish societal perspective, including the cost of interventions, additional health care, drugs, and production loss. Benchmark ICER thresholds vary and may be arbitrary.^[27]^ In Sweden ICERS over approximately US $60,000 (500,000 SEK) are considered to be high from a societal perspective.^[28]^ In the UK, interventions from a health care perspective (including health care costs only) can be considered cost-effective when a QALY gained costs less than approximately US $26,000 (£20,000).^[29]^ Thus, the results are also presented here from a health care perspective.

#### 2.4.2 Health-related quality of life

In order to calculate QALYs, the primary HRQoL measurement used in this study was the EQ-5D.^[20]^ For comparison, another generic measurement, the SF-6D,^[21]^ and 1 disease-specific measurement, the NDI,^[30]^ were used at baseline and 1 year post-inclusion.

The EQ-5D, (as recommended by the National Institute for Health and Clinical Excellence [NICE]^[29]^), contains 5 items describing the participant's current health state, and each response is graded from 1 (no problem) to 3 (severe problems). The British value set (−0.594-1, with 1 representing full health) was used.^[20]^ Permission to use the EQ-5D was obtained from the EuroQol Group Foundation.

The SF-6D is a classification system with 11 questions derived from the SF-36 questionnaire to be used in economic evaluations.^[21]^ Each question has between 4 and 6 response options, generating a value set from 0.296 to 1, with 1 representing full health. A license to use the SF-36 was obtained from Quality Metrics Inc., USA.

Both the EQ-5D and SF-6D come with sets of preference weights, called utility scores, to predict the values of different health states.^[31]^ Since disease-specific measurements are generally more responsive than generic measurements,^[32]^ converting a neck-specific measurement into a utility score has been proposed.^[24]^ High correlations between the disease-specific NDI and SF-6D in neck surgery reportedly permits the NDI to be used to calculate overall changes in utilities, and thus QALYs.^[24]^ The NDI is a reliable and valid measure^[25]^ consisting of 10 questions measuring neck related disability with each item scored on a scale from 0 (no disability) to 5 (severe disability).

#### 2.4.3 Production loss

Indirect costs mainly consist of production loss, that is, sick leave due to WAD. Data on sick leave due to neck disorders were collected from the Swedish Social Insurance Agency. Neck-related baseline sick leave was based on registry data on the number of days 6 months pre-inclusion. As short-term sick leave is generally paid by the employers in Sweden, these number of days were collected from patient questionnaires at 3, 6, and 12 months (post-inclusion) when not exceeding 14 days. For the purpose of generalization, the mean salary in Sweden according to Statistics Sweden (www.scb.se/en) was used. The cost of production loss was calculated using the human capital approach including gross salary plus taxes and 365 working d/y (US$ 165/d).

#### 2.4.4 Health care and drugs

Direct costs (i.e., health care costs) were determined from patient questionnaires at 3, 6, and 12 months, and from physiotherapist reports. Out-of-pocket costs for the patients were not available. The number of physiotherapist visits within the study was reported by the treating physiotherapists (n  =  69), who were also asked to estimate the number of patients that could be treated simultaneously in the gym with the intervention in question. Patient questionnaires included questions about additional health care not included in the interventions (caregiver and number of visits), use of analgesics, type, and dose.

Costs are based on county council price lists in Sweden for 2015 and are valued in US dollars (US $) for August 2016. Costs attributed to the intervention programs are based on costs for primary care physiotherapist visits ($83/visit). The average duration of a primary care physiotherapist visit is 35 minutes based on planned production by the Western County Council of Östergötland, Sweden. Intervention costs were based on the number and duration of visits divided by the number of patients estimated to be possible to treat simultaneously at each session. Costs for additional reimbursed health care are priced per visit as follows: general practitioner (GP) $259, physician at hospital; orthopedic $300, emergency (assuming office hours) $855, pain clinic $682, psychologist/counselor, $245, multi-professional investigation at pain clinic, $3438, multimodal treatment program at pain clinic, $6404, and chiropractor/naprapath, $126 (first)/42 (additional). Drug costs are based on pharmacy retail prices.

### 2.5 Data analysis

**Figure 2:**
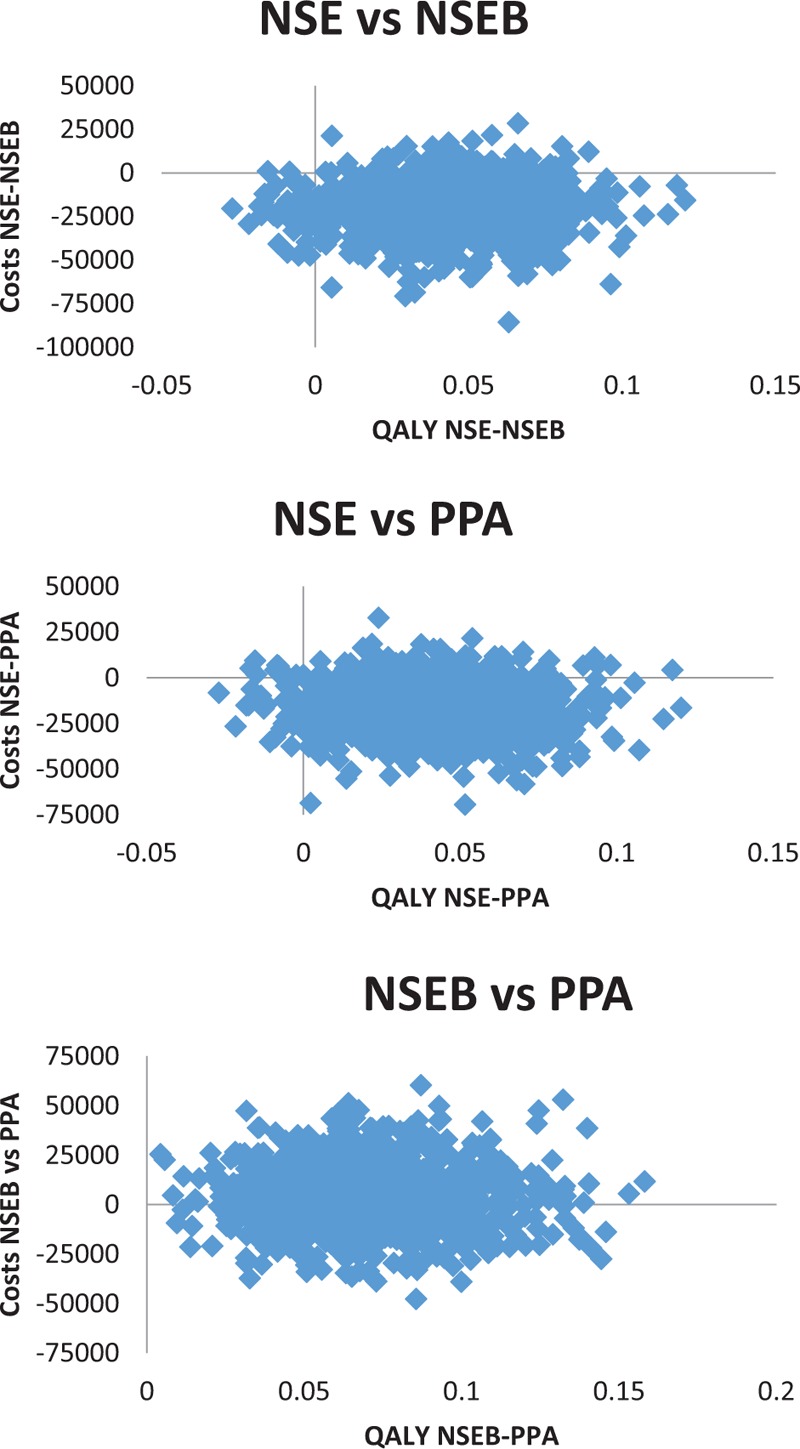
Cost-effectiveness planes of bootstrapped ratios. NSE  =  neck-specific exercise, NSEB  =  neck-specific exercise with a behavioral approach, PPA  =  prescription of physical activity, QALY  =  quality adjusted life year, based on Euroqol 5 Dimension Quality of life questionnaire.

The sample-size calculation for the main Randomized cntrolled trial,^[16]^ was based on the primary study outcome, the NDI (n  =  216). Between-group comparisons of normally distributed parametric data with similar variance were evaluated by one-way analysis of variances with Tukey correction for post-hoc tests. HRQoL scores were treated as parametric. Kruskal–Wallis with the post-hoc Mann–Whitney *U* test was used for ordinal scales and chi-square for bivariate outcomes. For derivation of costs, bootstrapping (1000 times) were used. Uncertainty was graphically illustrated by also plotting bootstrapped cost-effectiveness planes (Fig. 2). Independent samples *t* tests for parametric data or Mann–Whitney *U* test for ordinal data were used for dropout analyses. SPSS version 22 (SPSS Inc., Chicago, IL) was used for calculations and statistical significance was set at *P* < .05. SF-6D utility scores were obtained from SF-36 measurements using Quality Metrics scoring software, version 4.0. The NDI score was converted into an SF-6D utility score using a formula proposed by Richardson and Berven,^[24]^ −0135 × NDI + 0.8636. To explore the variance of the SF-6D and EQ-5D explained by the NDI in the current study sample, a linear regression model was used. Pearson/Spearman correlation tests were used for parametric/non-parametric correlations between HRQoL outcomes and age/sex to analyze the impact of baseline differences. QALYs (on a scale with anchor points 0 [death] and 1 [full health]) were calculated from the SF-6D, EQ-5D, and NDI assuming gradual linear change during the year studied. Thus, each utility change score was divided by 2 to get the average QALY-weight during the year analyzed. The analyses were made on an intention-to-treat basis, including all patients completing the follow-up. Discounting was not applied since the scope of this study was 1 year.

## 3 Results

### 3.1 Flow of participants, therapists, and centers through the study

The intervention groups did not differ in any of the baseline variables except the allocation of more women to the NSE group, which was also slightly younger (Table 1). However, there was no correlation between age (all r/rhos <0.13, *P* > .07) or sex and any of the outcomes (all r/rhos <0.16, *P* > .06). The 1-year follow-up was completed by 170 individuals (79%) (Fig. 1). Drop-outs were somewhat younger (mean age 37, SD 11 years) than those who completed the study (mean age 41, SD 11 years, *P*  =  .04), but there were no differences (all *P* > .26) between those who completed the questionnaires and drop-outs regarding baseline pain, allocation, sex, WAD grade, or any utility scores.

**Table 1:**
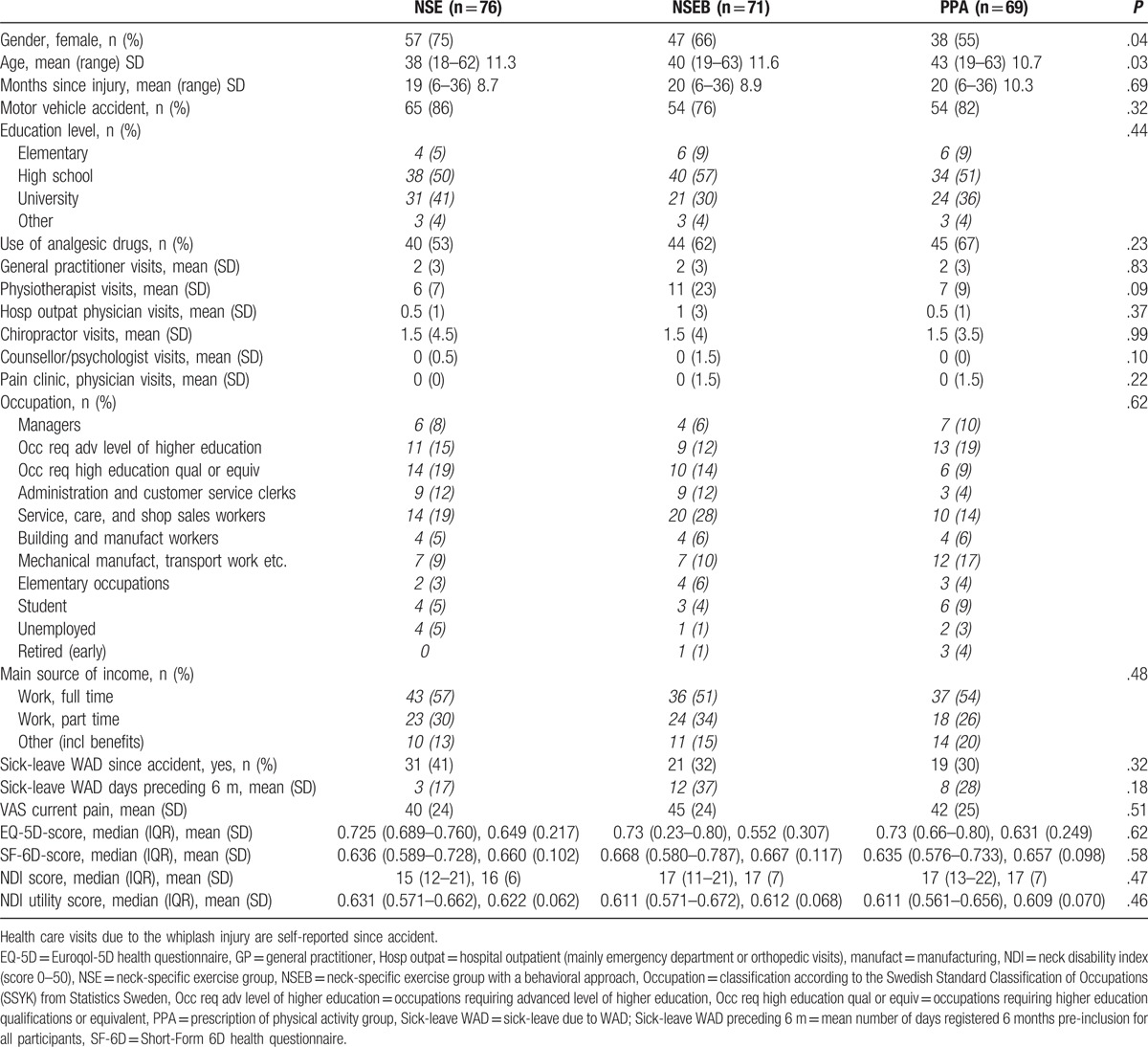
Background and baseline variables of participants with chronic whiplash-associated disorders (WAD).

### 3.2 Effectiveness: health-related quality of life

At 1 year, the NSEB group reported greater improvements in EQ-5D than the PPA group, which reported a deterioration of health (Table 2). This was also the case for the NDI utility scores for both NSE and NSEB versus PPA (Table 2). Both the NSE and NSEB groups reported greater improvements in 3 dimensions of the SF-6D (bodily pain, *P* < .01; physical functioning, *P* < .01; and social functioning, *P*  =  .02), but the difference in the SF-6D utility change score was not significant (Table 2). There was no significant difference in any of the outcomes between the NSE and NSEB groups.

**Table 2:**
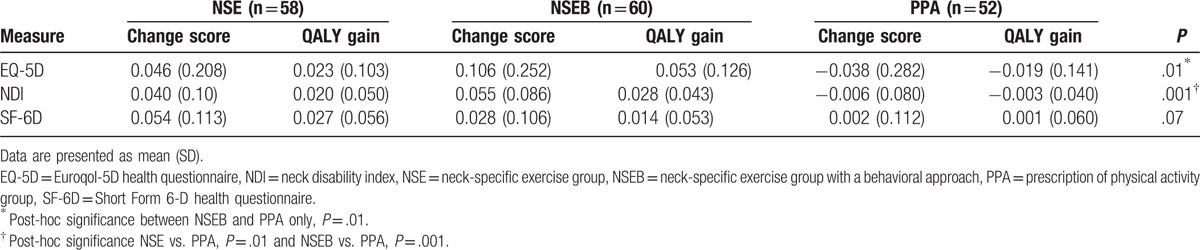
Utility change scores and QALY gains for different outcome measures after 1 year.

#### 3.2.1 Correlation between measurements

There was a moderate correlation between the 3 different utility scores (*r*; SF-6D and EQ-5D, 0.49; NDI and EQ-5D/SF6D, −0.53/−0.56; all *P* < .001). The NDI explained 31% of the variance in the SF-6D utility score and 40% of the EQ-5D score.

### 3.3 Costs

#### 3.3.1 Production loss

The NSE group tended to have the lowest cost of sick-leave for 1 year post-inclusion, but the difference between groups was not significant (Table 3). No participants received disability pension due to WAD at baseline, but 1 participant in the PPA group received a 50% disability pension due to WAD 6 months post-inclusion.

**Table 3:**
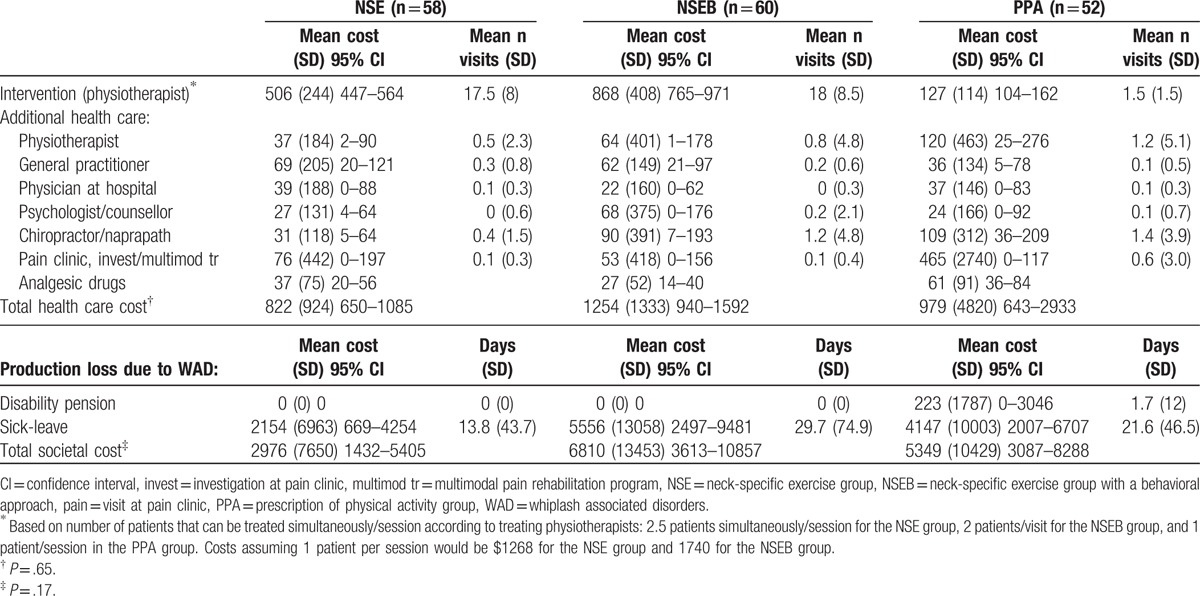
Health care and societal costs in US dollars 1 year following 3 exercise regimes in chronic whiplash associated disorders.

#### 3.3.2 Intervention costs and additional health care

The mean number of intervention and other health-care visits and costs are presented in Table 3. The mean number of patients that could be treated at the same time, as estimated by the physiotherapists, was 2.5 (SD1, NSE) or 2 (SD1, NSEB, range both groups 1–5). Though the PPA intervention was cheaper, the total health care cost was not (*P*  =  .53). Fewer participants in the 2 neck-specific groups reported using analgesics at 1 year,^[17]^ but no significant difference was found regarding the costs (Table 3).

### 3.4 Cost-utility

As no QALY gain occurred in the PPA group (Table 2), this intervention was considered non-relevant. Using the EQ-5D or NDI, there was a tendency for the NSEB group to be more effective than the NSE group, but the NSEB group was also more than twice as expensive from a societal perspective, mainly due to production loss (Tables 2 and 3). If using the SF-6D, the NSE group dominated the NSEB group, as NSE alone tended to be both more effective and less costly than the addition of NSEB. The ICERs for adding NSEB versus NSE alone were high above the thresholds from a societal perspective, but the sums varied depending on the chosen HRQoL outcome (Table 4, Fig. 2). The ICER from a health care perspective was reasonable however (Table 4). The intervention cost alone, per QALY gain in the NSE group was $12,067.

**Table 4:**
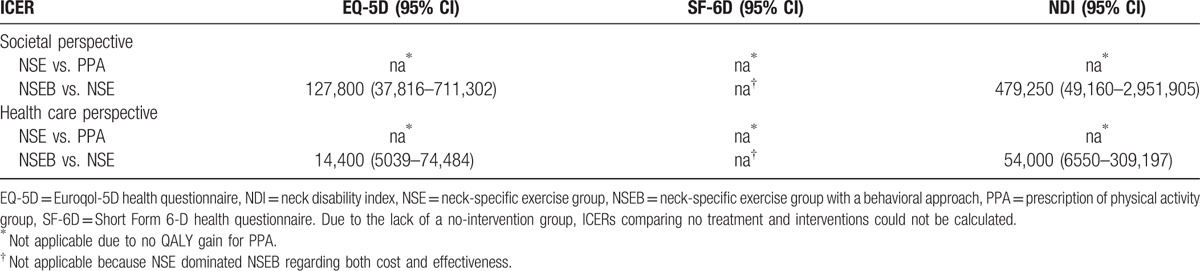
ICERs in US dollars per QALY from a societal and health care perspective comparing 3 interventions using 3 different health-related quality of life outcome measurements.

### 3.5 Sensitivity analyses

To account for uncertainties, cost-effectiveness planes were bootstrapped (Fig. 2) and a sensitivity analysis of the number of physiotherapy visits was performed. The ICERs for adding NSEB versus NSE alone from a societal perspective ranged from $118,867 to 131,467 (5–1 patients/session), and from a health care perspective from $5467 to 18,067.

## 4 Discussion

QALY gains from baseline to the 1-year follow-up were reported for both physiotherapist-led neck-specific exercise interventions, but no gain was reported for the PPA intervention which was thus a non-relevant option. The gains were different depending on the HRQoL measurement used, but regardless of measurement, the NSE intervention was the cost-effective intervention from a societal perspective. Even though there was a tendency for the NSEB group to have larger QALY gains using the EQ-5D or NDI, the societal cost in the NSEB group was higher than in the NSE group. The additional costs for adding a behavioral approach per QALY gained (i.e., ICERs) were substantially higher than the threshold defining cost-effectiveness.^[28,29]^ However, from a health care perspective, the ICERs between NSE and NSEB were reasonable, unless using the SF-6D. If using the SF-6D the NSE intervention was both cheaper and tended to be more effective from both perspectives. Production loss was the largest societal cost, which is consistent with previous reports.^[3]^ It should, however, be acknowledged that costs for production loss may be lower if using the friction cost approach rather than the human capital approach used in this study.^[33]^

Even though the 2 physiotherapist-led interventions were more expensive than PPA, they tended to generate less additional care and the total health care costs for the PPA group was thus not lower. The total costs for the interventions were lower (NSE) or just slightly more expensive (NSEB) than 1 single physician visits at an emergency department during office hours, or less than 15% of the price of a multimodal treatment program at a pain clinic. Unpublished registry data for hospital visits from 2 participating regions indicate that approximately 30% of all neck-related physician visits at hospitals by this chronic WAD sample during the 1-year follow-up period, were at an emergency department. Since participants were relatively young there are potentially larger sums to be saved over time.

The results in this study of people with chronic WAD grade 2 to 3 differ from those reported in the acute phase.^[3]^ This is most likely due to the fact that the recovery rate after a whiplash trauma, especially in lower WAD grades (as included in the acute study), is high^[4]^ and a more extensive treatment program may not be needed on a group level, and is thus not considered cost-effective.^[3]^ However, spontaneous improvement is unlikely in people with chronic WAD, and neck-specific exercise is more effective than PPA in the treatment of chronic WAD grade 2 to 3.^[16–18]^

The intervention costs were based on the physiotherapists’ estimations of the average number of patients that they could treat at the same time. Depending on gym capacity, the personal needs of the patients, the availability of other patients that can be treated at the same time, and the personal skills and wishes of the physiotherapists, this number can vary (estimated range 1–5). However, the sensitivity analysis, varying the number of patients/session, did not change the conclusion. Since no other exercise studies including chronic WAD grade 3 have been published, long-term effects over many years are unknown, and a Markov Chain model was not considered to be useful.

Both the EQ-5D and SF-6D come with sets of preference weights obtained from the general population, which enables comparisons between different groups of patients. However, patients and the general population may interpret health state descriptions differently.^[34]^ The ability to use disease-specific measures for QALY calculations reportedly allows the use of more responsive outcome measures, while also allowing comparisons for various treatment options.^[24]^ However, the algorithm used for the NDI does not allow utility scores to be generated in the very upper or lower ranges (>0.88 or <0.2).^[24]^ This ceiling effect was seen in 7 individuals at 1 year only, but no floor effect was observed at either time point. In our sample, the variance in SF-6D score explained by the NDI was much less (*R*^2^  =  0.31) than in the sample awaiting spinal surgery tested by Richardson and Berven (*R*^2^  =  0.71). This difference may indicate that the NDI may be less appropriate for QALY calculations in chronic WAD than in spinal surgery or that other formulas are needed. Nonetheless there was no significant difference between the scores or score changes (EQ-5D, SF-6D, NDI) unless analyzing 1 intervention group at a time. The change score generated by the EQ-5D was significantly higher than the score generated by the SF-6D in the NSEB group. This relationship between EQ-5D and SF-36 has also been reported in the treatment of rheumatoid arthritis.^[35]^ Because the NSEB group had the lowest mean baseline EQ-5D score, our findings are in accordance with previous reports that the EQ-5D is more sensitive to changes in lower scores^[23]^ and demonstrate the importance of considering the impact of the HRQoL measurement used. Thus, caution is warranted when comparing cost-utility between studies using different HRQoL outcomes.

The costs of additional health care and use of drugs are somewhat uncertain because they are self-reported, and even though participants were asked 4 times during the study year, recall bias cannot be ruled out and the doses/number of visits may be inexact. In addition, some drugs (e.g., paracetamol) are available without a prescription, making these costs to society somewhat uncertain. However, from a societal perspective, additional health care costs and drugs had a low overall impact, and excluding these costs from the analysis did not alter the results. Out of pocket costs incurred by the participants and patient time were not available. However adding these costs would not have changed the results, since the only anticipated difference between the 2 groups with QALY gains (NSE/NSEB) was more patient time for the NSEB group, where the ICER compared with the NSE was already above the threshold.

Even though NSE generated lower costs per QALY gain, NSEB or PPA may still be cost-effective to certain individuals. The NSEB group also tended to have more sick-leave at baseline, which may have had an impact on the continuous level of sick-leave. Furthermore, due to the lack of a no-intervention control group, the cost-effectiveness in this study only relates to the 3 study interventions, not to no treatment at all. However, as changes in symptoms after 6 months are unlikely, QALY gains without any intervention are not likely.

To summarize, HRQoL improved following physiotherapist-led neck-specific exercise either with or without a behavioral approach. However, the addition of a behavioral approach was not cost-effective compared with neck-specific exercise alone. The prescription of physical activity did not result in any QALY gain and was thus not considered a relevant option.

## Acknowledgments

The authors thank all of the participants in this study, including WAD participants, physiotherapists, and staff involved at any stage of the study.
