# Cost-effectiveness of neck-specific exercise with or without a behavioral approach versus physical activity prescription in the treatment of chronic whiplash-associated disorders: Analyses of a randomized clinical trial: Erratum

**DOI:** 10.1097/MD.0000000000007730

**Published:** 2017-08-04

In the article, “Cost-effectiveness of neck-specific exercise with or without a behavioral approach versus physical activity prescription in the treatment of chronic whiplash-associated disorders: Analyses of a randomized clinical trial”,^[1]^ Dr. Gunnel Peterson should just have PhD listed and Anneli Peolsson should have Professor, PhD listed in the byline.
